# Reduced Contraction of Blood Clots in Venous Thromboembolism Is a Potential Thrombogenic and Embologenic Mechanism

**DOI:** 10.1055/s-0038-1635572

**Published:** 2018-03-28

**Authors:** Alina D. Peshkova, Dmitry V. Malyasyov, Roman A. Bredikhin, Giang Le Minh, Izabella A. Andrianova, Valerie Tutwiler, Chandrasekaran Nagaswami, John W. Weisel, Rustem I. Litvinov

**Affiliations:** 1 Department of Biochemistry and Biotechnology, Institute of Fundamental Medicine and Biology, Kazan Federal University, Kazan, Russian Federation; 2 Department of Vascular Surgery, Inter-Regional Clinical Diagnostic Center, Kazan, Russian Federation; 3 Department of Cell and Developmental Biology, University of Pennsylvania School of Medicine, Philadelphia, Pennsylvania, United States

**Keywords:** blood clotting, thrombosis, clot contraction, clot retraction, venous thromboembolism

## Abstract

Contraction (retraction) of the blood clot is a part of the clotting process driven by activated platelets attached to fibrin that can potentially modulate the obstructiveness and integrity of thrombi. The aim of this work was to reveal the pathogenic importance of contraction of clots and thrombi in venous thromboembolism (VTE). We investigated the kinetics of clot contraction in the blood of 55 patients with VTE. In addition, we studied the ultrastructure of ex vivo venous thrombi as well as the morphology and functionality of isolated platelets. Thrombi from VTE patients contained compressed polyhedral erythrocytes, a marker for clot contraction in vivo. The extent and rate of contraction were reduced by twofold in clots from the blood of VTE patients compared with healthy controls. The contraction of clots from the blood of patients with pulmonary embolism was significantly impaired compared with that of those with isolated venous thrombosis, suggesting that less compacted thrombi are prone to embolization. The reduced ability of clots to contract correlated with continuous platelet activation followed by their partial refractoriness. Morphologically, 75% of platelets from VTE patients were spontaneously activated (with filopodia) compared with only 21% from healthy controls. At the same time, platelets from VTE patients showed a 1.4-fold reduction in activation markers expressed in response to chemical activation when compared with healthy individuals. The results obtained suggest that the impaired contraction of thrombi is an underappreciated pathogenic mechanism in VTE that may regulate the obstructiveness and embologenicity of venous thrombi.

## Introduction

Deep vein thrombosis (DVT) of the lower limbs, either isolated or associated with pulmonary embolism (PE), together known as venous thromboembolism (VTE), complicates many diseases and remains one of the most medically and socially challenging disorders due to the relatively high incidence of life-threatening complications. Collectively, DVT and PE are among the leading causes of death from cardiovascular diseases. 1 In the United States, there are more than 200,000 new cases of VTE registered annually; ∼10% of these cases are associated with PE. Mortality during the first 3 months in patients with PE varies from 1.4 to 17.4%. 2 3 Moreover, VTE is associated with long-term complications; in fact, at 3 years post-VTE, 35 to 70% of patients have a disability caused by chronic venous insufficiency and other consequences of the post-thrombotic syndrome. 3 Despite numerous studies on VTE, the causes and pathogenic mechanism of DVT and PE are not fully understood and the prevention and treatment of VTE remain unsatisfactory. 4

Virchow's triad characterizes the most general causes of VTE as a combination of blood hypercoagulability, changes of blood rheology, and local damage to the endothelium. 5 6 7 However, the molecular and cellular mechanisms underlying these abnormalities remain largely unclear. Blood cells contribute substantially to the pathogenesis of VTE. 8 9 10 Venous thrombi are known to be rich in fibrin but also to contain activated platelets, platelet aggregates, red blood cells (RBCs), and white blood cells. The presence of white blood cells solidifies the connection of venous thrombosis with local inflammation. 8 11

The role of platelets is perhaps one of the least explored aspects of VTE; however, there is evidence to suggest that platelets may be involved not only in clot formation but also in the growth and remodeling of a thrombus. 12 13 A blood clot undergoes spontaneous volumetric shrinkage, which is known as clot contraction or retraction. This process is driven by platelet contractile proteins, actin and myosin, which form an active complex that generates a mechanical force through a molecular mechanism similar to muscle contraction or cellular motility. 14 15 16 Although the role of the volume shrinkage of clots and thrombi in vivo is largely unknown, it has been implicated in the restoration of blood flow past otherwise obstructive thrombi. 12 17 18

Despite the potential clinical importance of contraction of blood clots and thrombi, a systematic study of this process has not been performed in patients with venous thrombosis. The aim of this study was to determine a possible pathogenic role and clinical significance of contraction of blood clots in VTE by studying the kinetics of clot contraction in the blood of patients with VTE in association with clinical characteristics, platelet function, and laboratory parameters.

## Materials and Methods

### Patients, Inclusion, and Exclusion Criteria

The study was approved by the Ethical Committee of the Interregional Clinical Diagnostic Center (Kazan, Russian Federation) and informed written consent was obtained from the VTE patients and healthy donors. Fifty-five patients with VTE were enrolled in the study based on the inclusion and exclusion criteria listed in the Supplementary Material , which is ∼10% of all patients admitted to the emergency room of the Department of Vascular Surgery of the Interregional Clinical Diagnostic Center (Kazan, Russian Federation) during 2014–2016 (see flowchart for exclusion criteria in the Supplementary Material ). The group of studied patients had the same basic characteristics as the overall cohort ( Supplementary Table S1 ). The main clinical subgroups are presented in Table 1 . DVT was verified by duplex ultrasonography of the vessels using the GE Logiq 700 EXP unit. Patients with suspected PE underwent a CT scan of the chest with contrast. This procedure was performed in 32 patients, of whom 23 (71%) were diagnosed with PE. An embolus occluding >50% of the pulmonary artery (massive PE) occurred in 2 (9%) patients; occlusion of 30 to 50% of the vascular bed (sub-massive PE) occurred in 8 (35%), and only small branches of the pulmonary artery (<30% of the vascular bed) were occluded in 13 (56%) patients.

**Table 1:** Clinical characteristics of patients with VTE

Characteristics of thrombosis ( *n* = 55)		
Floating part of a thrombus	>7 cm	4 (7%)
	<7 сm	26 (47%)
	No floating part	25 (46%)
Thrombosis	Provoked	8 (15%)
	Unprovoked	47 (85%)
Duration of symptoms	>21 d (subacute)	10 (18%)
	<21 d (acute)	45 (82%)
Comorbidities ( *n* = 52) a		
Malignant neoplasms		7 (14%)
Acute infections		1 (2%)
Thrombosis of superficial veins		14 (26%)
Cardiac ischemia		5 (10%)
Hypertension		20 (38%)
Diabetes		5 (10%)
Risk factors ( *n* = 78) b		
History of varicose disease of the lower extremities		19 (24%)
History of acute disorders of cerebral circulation		4 (5%)
Extensive trauma		4 (5%)
Immobilization for more than 4 d		5 (6%)
Prior surgery (within 4 wk)		2 (3%)
History of VTE		8 (11%)
Thrombophilia		3 (4%)
Smoking		20 (25%)
Obesity (BMI > 30 kg/m ^2^ )		11 (15%)
Oral contraception		1 (2%)

Abbreviations: BMI, body mass index; VTE, venous thromboembolism.

a The number of comorbidities (52) is less than the total number of patients with thrombosis (55) because not all of them had identified comorbidities.

b The number of risk factors (78) is more than the total number of patients with thrombosis (55) because some patients had more than one risk factor.

It is important to note that VTE patients were excluded from this study if they were given any anticoagulants (direct or indirect), thrombolytics, and antiplatelet drugs (including nonsteroidal anti-inflammatory drugs) within 1 month prior to examination. Only one patient had stopped taking baby aspirin for 7 days prior to enrollment, but his platelet aggregation was normal. These patients were not on medication as they refrained from seeking medical attention until the manifestation of profound clinical symptoms such as pain and leg swelling. These rigorous exclusion criteria ensured that all patients enrolled in this study were free of specific drugs that could potentially influence blood clotting or platelet function and resulted in only 55 patients being enrolled out of the >550 VTE patients admitted to the Department of Vascular Surgery of the Interregional Clinical Diagnostic Center (Kazan, Russian Federation) over a 2-year period of this study (2014–2016).

Of the 55 patients in the study, 5 patients were diagnosed with coronary heart disease, 4 patients had previously had an ischemic stroke and presented with minor neurological deficits, 7 patients previously had cancer (uterus—2, mammary gland—2, prostate—1, gastric—1). All patients who were previously prescribed aspirin affirmed that they no longer took the prescription and cancer patients who had previously undergone surgery were no longer receiving any cancer treatments. Eighteen patients were on antihypertensive medications (β-blockers, angiotensin receptor blockers, and ACE inhibitors). Eight of the patients enrolled in the study had provoked VTE (hip replacement surgery—2, acute trauma—4, long-term immobilization—2).

The control group consisted of 60 aspirin-free healthy donors who comprised a similar age range and gender percentage as the patient population. The control group was made up of 37 (62%) men and 23 (38%) women with an average age of 51 ± 2 years. This group closely matched the group of VTE patients, which was composed of 37 (67%) men and 18 (33%) women with an average age of 55 ± 2 years.

### Blood Collection and Processing

Blood collection and handling was performed in accordance with the approved guidelines and based on the preanalytical requirements. 19 Venous blood was drawn at the time of the patient's admission to the emergency room and healthy donors donated blood at the Department of Blood Transfusion. Blood was collected into vacutainers containing 3.8% trisodium citrate 9:1 by volume and analyzed within 4 hours. One whole blood sample was used for the clot contraction assay. A portion of the blood was centrifuged (2,500 *g* , 15 minutes) to obtain platelet-poor plasma that was used for blood coagulation tests. A portion of the whole blood was reserved for platelet isolation. Another blood sample was stabilized with K _3_ -EDTA and used for hematological tests. A nonstabilized whole blood sample was mixed with a clotting activator and allowed to clot for 20 to 30 minutes at 37°C, followed by centrifugation (2,500 *g* , 15 minutes) to obtain serum for biochemical blood tests.

### Platelet Isolation

Fresh citrated blood of 11 randomly selected VTE patients or 11 healthy donors was centrifuged at 200 *g* for 10 minutes at room temperature with a light brake to obtain platelet-rich plasma. Isolated platelets were collected in the void volume after gel filtration of the platelet-rich plasma on Sepharose 2B equilibrated with Tyrode's buffer (4 mM HEPES, 135 mM NaCl, 2.7 mM KCl, 2.4 mM MgCl _2_ , 5.6 mM D-glucose, 3.3 mM NaH _2_ PO _4_ , 0.35 mg/mL bovine serum albumin, pH 7.4). Cell viability was ∼97% based on the maintenance of the mitochondrial membrane potential (ΔΨm) determined by flow cytometry using a ΔΨm-sensitive fluorescent dye MitoTracker DeepRed FM (Invitrogen, Carlsbad, California, United States). Platelet count was performed in a hemocytometer. Platelets were used within 3 hours of blood collection.

### Scanning Electron Microscopy of Venous Thrombi

Of the 55 patients enrolled in this study, 9 underwent thrombectomy in accordance with the National Russian Guidelines 20 that recommend thrombectomy in embologenic thrombosis; the floating portion of the thrombus is considered embologenic when it is ∼3 cm in size and protruding into the femoral or iliac vein or the inferior vena cava. Six patients had a thrombectomy of the common femoral vein and three in the common femoral and iliac veins. Fresh thrombi extracted during thrombectomy were fixed in 2% glutaraldehyde. The fixed clots were washed in 50 mM sodium cacodylate with 100 mM NaCl (pH 7.4), then dehydrated and sputter-coated. The thrombi were cut-open and the interior parts of thrombi were examined in a FEI Quanta 250FEG scanning electron microscope (FEI, Hillsboro, OR). For each thrombus, 10 to 15 high magnification micrographs were analyzed taken at randomly chosen locations.

### Continuous Optical Tracking of Contracting Blood Clots in Vitro

**Fig. 1:**
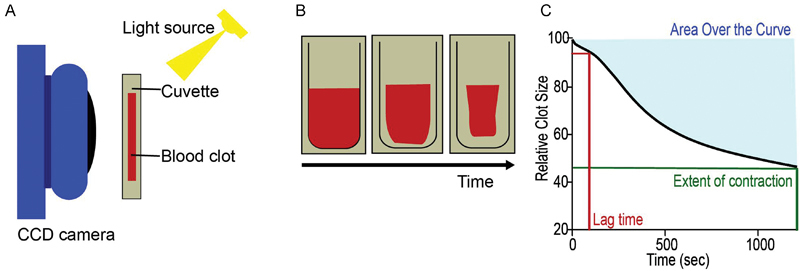
Schematic of experimental methods. ( **A** ) Recalcified whole blood samples with thrombin were added to a cuvette and allowed to contract. Images were recorded by a charge-coupled device camera every 15 seconds based on the light scattering properties of the clot versus the expelled serum. ( **B** , **C** ) Data on relative clot size were recorded over time, where the original kinetic curve could be analyzed for the extent of clot contraction (green) at 20 minutes, the lag time (red) or the time to 5% contraction, or the area over the curve (blue), representing the work done by the contracting clot.

In the blood of all 55 VTE patients and 60 healthy individuals, determination of the kinetics and the extent of clot contraction was completed using the previously described method based on the optical detection of clot size over time using the Thrombodynamics Analyzer System (HemaCore, Russian Federation). 13 Citrated blood samples from patients and healthy donors were activated with 1 U/mL human α-thrombin (Sigma-Aldrich, St. Louis, Missouri, United States) and 2 mM CaCl _2_ (final concentrations). Activated samples (80 µL) were quickly transferred to a 12 × 7 × 1 mm transparent plastic cuvette that was precoated with a thin layer of 4vol% Triton X-100 in phosphate-buffered saline to prevent sticking of the clot to the chamber without affecting the clot structure and platelet functionality. The transparent cuvette was placed into the 37°C temperature-controlled chamber of the Thrombodynamics Analyzer instrument. The cuvette had two compartments and the experiments were performed simultaneously in duplicate. Images of the clots were taken every 15 seconds for 20 minutes to track the changes in the relative clot size (the overall portion of the cuvette containing the clot) based on the light scattering. The collected images were analyzed computationally to extract the following parameters of clot contraction: the extent of contraction (calculated as [( *S*_0_ – *S _t_ )/S *_0_ ] × 100, where *S*_0_ is the initial clot size and *S _t_* is the final clot size at the end point *t* = 20 minutes); lag time (time from the addition of thrombin until the clots reaches 95% of its initial size); the average contraction velocity (%/s); and the area over the kinetic curve limited by a horizontal line at the initial contraction point on the *y* -axis roughly corresponding to the amount of mechanical work on clot compression done by the contracting platelets ( Fig. 1 ).

### Coagulation, Hematological, and Biochemical Tests

In all 55 VTE patients and 60 healthy individuals, an automated coagulometer Sysmex CA-1500 (Sysmex, Canada) was used with fresh citrated plasma samples for the following tests: activated partial thromboplastin time (aPTT), prothrombin time, INR, fibrinogen, antithrombin III, and D-dimer concentrations. Platelet aggregation was studied using an optical aggregometer (Biola, Russia), where platelets were activated with 20 μM ADP (Renam, Russia). Cell count was performed in EDTA-treated whole blood samples with an ABX Pentra 60 cell counter (hematology analyzer; Horiba, Japan). The following parameters were analyzed: erythrocyte count, mean corpuscular volume, hematocrit, hemoglobin, mean corpuscular hemoglobin, color index, leukocyte count, monocyte count, neutrophil count, lymphocyte count, eosinophil count, basophil count, platelet count, and mean platelet volume. Blood biochemistry tests were performed with RX Imola (Randox, UK) and Advia 1200 (Siemens, Germany) analyzers (Siemens, Germany).

### Flow Cytometry of Quiescent and Activated Platelets

Platelet functionality was analyzed in 11 VTE patients and 11 healthy individuals in parallel by expression of P-selectin (CD62p) and active integrin αIIbβ3 (determined by its fibrinogen-binding capacity) before and after activation with a thrombin-receptor activation peptide (TRAP-6, the PAR-1-specific hexapeptide Ser-Phe-Leu-Leu-Arg-Asn; Bachem Americas Inc., Torrance, California, United States). TRAP-6 was added to isolated platelets at 50 µM for 3 minutes at room temperature. Then platelets (200,000 in 50 µL) were incubated for 10 minutes with anti-human-CD62p phycoerythrin-labeled murine antibodies (BD Biosciences, San Jose, California, United States) (0.045 µg/mL) or Alexa fluor 488-labeled human fibrinogen (ThermoFisher Scientific, Waltham, Massachusetts, United States) (5 µg/mL). After incubation with the labeled ligands, the platelets were analyzed using a FacsCalibur flow cytometer equipped with BD CellQuest software. Platelets were gated based on their size and granularity and 5,000 platelets were counted in each sample. FlowJo X software was used for data analysis.

### Scanning Electron Microscopy of Platelets

Isolated platelets (1,000,000 in 100 µL of phosphate-buffered saline, pH 7.4) obtained from four VTE patients and four healthy donors were processed in parallel and fixed in 2% glutaraldehyde for 90 minutes at room temperature. Fixed platelets were layered on a carbon filter (0.4 µm pore size) and centrifuged at 150 *g* for 7 minutes. The samples were then rinsed three times with the phosphate-buffered saline, dehydrated in 30 to 100vol% ethanol, and dried with hexamethyldisilazane. The samples were sputter-coated with gold palladium. Micrographs were taken with a scanning electron microscope Merlin (Zeiss, Germany). Not less than 10 randomly selected images were analyzed from each platelet preparation and a total of 4,211 and 480 platelets were analyzed from VTE patients and healthy individuals, respectively.

### Statistical Analysis

Statistical analysis was done using Prism GraphPad 6.0. Data were analyzed for statistical significance using a two-tailed unpaired *t* -test with a 95% confidence level ( *p* = 0.05). This type of analysis was performed to compare the clot contraction laboratory parameters between the clinical groups and subgroups. A chi-square test was applied to analyze the relative incidence qualitative morphological features of activated versus unactivated platelets in VTE patients and healthy donors with a 95% confidence level. Nonlinear regression analysis for contraction phases was completed using a piece-wise function to fit the three phases of contraction; the rate or rate constant was compared from each phase by a two-tailed *t* -test with a 95% confidence level. The results are presented as mean ± standard error of the mean (SEM) unless otherwise indicated.

## Results

### Evidence for Contraction of Venous Thrombi Occurring In Vivo

**Fig. 2:**
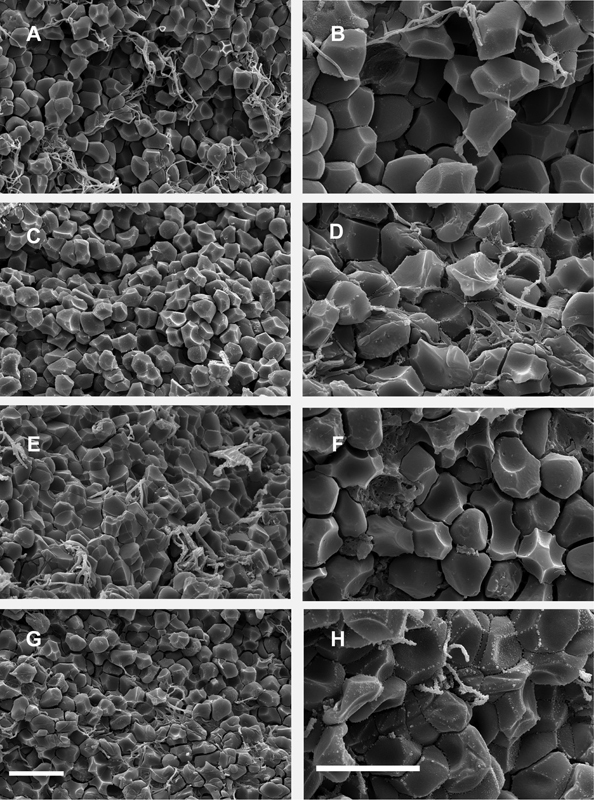
Representative scanning electron micrographs of the interior of venous thrombi containing compressed polyhedral erythrocytes (polyhedrocytes) as a morphologic signature of contraction in vivo. The corresponding images were taken from four randomly selected thrombi at lower ( **A** , **C** , **E** , **G** ) and higher magnification ( **B** , **D** , **F** , **H** ). Magnification bars = 10 µm.

It was recently shown that contraction of blood clots in vitro results in the deformation of RBCs to polyhedral shapes or polyhedrocytes. 12 Based on these observations, formation of compressed polyhedrocytes can be considered as the objective morphological criteria of clot contraction. Moreover, these morphological alterations could be used as a proof of the intravital compression of ex vivo clots and thrombi. 21 22 23 We analyzed the cellular composition of venous thrombi extracted from selected patients during interventional thrombectomy and found that polyhedrocytes comprised approximately one-half of all RBCs within thrombi ( Fig. 2 ). In addition, we observed intermediate forms between biconcave RBCs and fully formed polyhedrocytes, so that deformed (polyhedral and intermediate shape) RBCs together accounted for 80% of RBCs entrapped in venous thrombi. These results suggest the occurrence of intravital compression or contraction of venous thrombi, which may be a pathogenic mechanism of blood flow modulation at the sites of thrombotic occlusion. However, because of the selection criteria for thrombectomy, the extent of contraction in thrombi may differ in other VTE patients.

### Characterization of Clot Contraction in VTE Patients

**Fig. 3:**
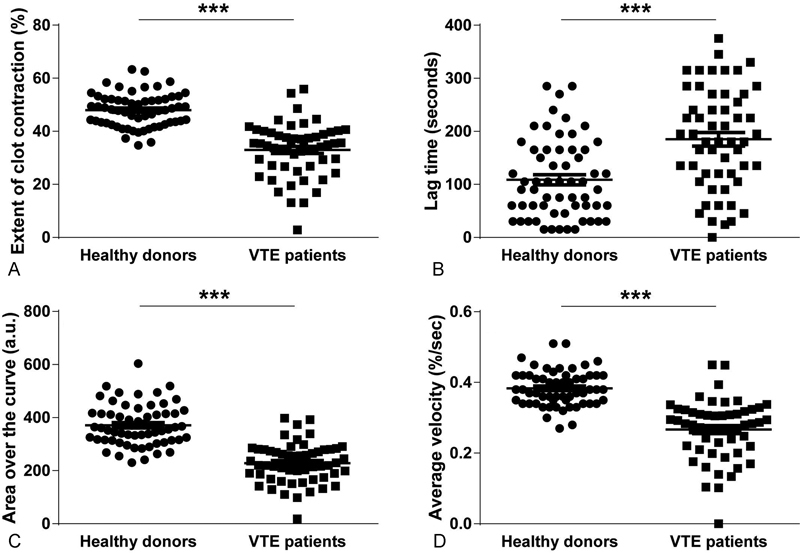
Clot contraction parameters of clots made from the blood of VTE patients ( *n* = 55) versus healthy donors ( *n* = 60). ( **A** ) Extent of clot contraction at 20 minutes, ( **B** ) lag time or time to reach 95% of the initial clot size, ( **C** ) area over the curve (an integral parameter that characterizes the intensity of the entire process of clot contraction), ( **D** ) average velocity of contraction.*** *p* < 0.001.

**Fig. 4:**
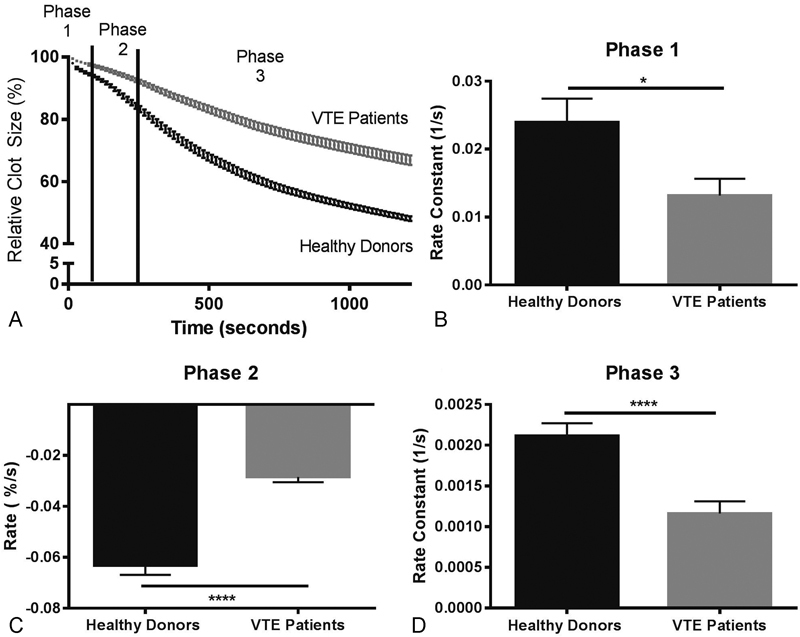
( **A** ) Averaged kinetic clot contraction curves obtained from clotting of the blood of VTE patients ( *n* = 55) and healthy individuals ( *n* = 60). Optical tracking was used to measure the relative changes in clot size over 20 minutes at 15-second intervals. ( **B** – **D** ) The rate constants of the three phases of clot contraction curves shown in **A** . The three phases correspond to the initiation of contraction (phase 1), linear contraction (phase 2), and mechanical stabilization (phase 3). Data are shown as mean ± SEM. * *p* < 0.5; **** *p* < 0.0001.

Despite the standardized clot initiation and platelet activation methodology, clots formed from the blood of patients with VTE contracted significantly slower and to a lesser extent than clots formed from the blood of healthy donors ( Fig. 3 , Supplementary Table S2 ). Specifically, there was a ∼1.5-fold reduction in the average velocity (0.26 ± 0.01%/second vs. 0.4 ± 0.01%/second, *p* < 0.0001), degree of clot contraction (33 ± 1% vs. 48 ± 1%, *p* < 0.0001), and area over the kinetic curve (228 ± 10 a.u. vs. 371 ± 10 a.u., *p* < 0.001), as well as an increase in the lag time (185 ± 13 second Vs. 108 ± 9 second, *p* < 0.001), in patients with VTE compared with healthy individuals, respectively. It has been previously shown that clot contraction occurs in three phases: initiation of contraction (phase 1), linear contraction (phase 2), and mechanical stabilization (phase 3). 13 Regression analysis conducted on average kinetics curves ( Fig. 4A ) revealed that in VTE patients and healthy donors all three phases were significantly different as indicated. The rate or rate constants of all three phases were significantly reduced in VTE patients compared with healthy individuals, indicating impairment of the mechanisms of contraction initiation, compaction, and stabilization of the clots ( Fig. 4B – D ).

### Relationship of Clot Contraction Parameters to Laboratory Tests

To examine if the changes in clot contraction were related to variations in hemostatic parameters, we analyzed the most remarkable VTE-related alterations in the blood tests ( Table 2 ). In VTE patients, there was a near 30-fold increase in D-dimer levels (6.6 ± 1.7 µg/mL vs. 0.23 ± 0.03 µg/mL, *p* < 0.001) associated with a relatively high fibrinogen level (3.9 ± 0.3 g/L vs. 2.7 ± 0.1 g/L, *p* < 0.001). At the same time, the rate of ADP-induced platelet aggregation was decreased in VTE patients (21.9 ± 7.5%/minute vs. 36.6 ± 1.0%/minute, *p* < 0.01), suggesting reduced sensitivity to platelet activators. Partial platelet refractoriness in combination with increased levels of fibrinogen in the blood are likely to contribute to the reduced ability of blood clots to contract in patients with VTE, as observed in ischemic stroke. 18 There was no strong parallelism between the changes in parameters of clot contraction and other laboratory tests (shown in Table 2 ), which indirectly points to the role of local reactions for contractility of clots and thrombi.

**Table 2:** Laboratory test results in VTE patients and healthy donors

Parameters (normal ranges are shown in parentheses)	VTE patients ( *n* = 55)	Healthy individuals ( *n* = 60)
Hemostatic parameters		
aPTT (26–36), s	32.5 ± 0.8	32.1 ± 0.5
Prothrombin ratio (70–130), s	95.5 ± 3 *	103.6 ± 1.6
Fibrinogen (1.8–4.0), g/L	3.9 ± 0.3 ***	2.7 ± 0.1
Thrombin time (14–21), s	16.8 ± 1.0	17.7 ± 0.2
D-dimer (0–0.5), µg/mL	6.6 ± 1.7 ***	0.23 ± 0.03
Antithrombin III (80–120), %	90.2 ± 8.8	90.5 ± 1.0
Prothrombin time (9.8–12.1), s	12.4 ± 0.9	11.1 ± 0.1
Maximal ADP-induced platelet aggregation (60–90), %	55.9 ± 15	64.3 ± 1.6
Rate of ADP-induced platelet aggregation (30–45), %/min.	21.9 ± 7.5 **	36.6 ± 1.0
Hematological parameters		
Platelet count (180–320), ×10 ^9^ /L	275 ± 16	286 ± 10
Red blood cells (3–5), ×10 ^12^ /L	4.3 ± 0.1 *	4.5 ± 0.1
Hematocrit (36–48), %	38 ± 0.1	39.5 ± 0.6
Hemoglobin (120–160), g/L	130 ± 4 ***	146 ± 2
Color index (0.85–1.05)	0.92 ± 0.02	0.93 ± 0.01
Mean cell volume (80–100), fL	89.9 ± 2.0 *	85.6 ± 0.8
Mean cell hemoglobin (30–35), pg	31.7 ± 1.1	32.1 ± 0.4
Leukocytes (4–9), ×10 ^9^ /L	8.7 ± 0.4 ***	5.8 ± 0.2
Eosinophils (0.5–5), %	2.7 ± 0.2	3.0 ± 0.3
Monocytes (3–11), %	7.7 ± 0.4 ***	6.2 ± 0.2
Lymphocytes (19–37), %	23 ± 1 ***	34 ± 4
Basophiles (0–1), %	0.68 ± 0.1	0.4 ± 0.1
Neutrophils (47–78), %	66.3 ± 1.0 *	58.8 ± 1.1
RDW (11–14.8), %	13.3 ± 0.4 *	14.2 ± 0.1
Mean platelet volume (8.6–12.6), fL	7.8 ± 0.1 *	9.4 ± 1.4
Biochemical tests		
Protein (60–83), g/L	70.2 ± 0.8 ***	72.8 ± 0.5
Bilirubin (5–17), µmol/L	13.0 ± 1.1 ***	8.4 ± 0.3
Creatinine (53–97), µmol/L	84.7 ± 2.3	87.2 ± 1.6
Urea (2.4–6.4), mmol/L	5.3 ± 0.2 **	4.4 ± 0.2
Cholesterol (3.6–5.2), mmol/L	4.8 ± 0.3	4.6 ± 0.1
ALT (5–40), U/L	29.4 ± 3.4 *	20.6 ± 1.3
AST (10–36), U/L	27.7 ± 2.6 ***	17.6 ± 0.7
Glucose (3.6–6), mmol/L	6.2 ± 0.2 ***	5.1 ± 0.1

Abbreviations: ALT, alanine aminotransferase; aPTT, activated partial thromboplastin time; AST, aspartate aminotransferase; RDW, red cell distribution width; VTE, venous thromboembolism.

* *p* < 0.05

** *p* < 0.01

*** *p* < 0.001.

### Analysis of Platelet Function and Morphology in VTE Patients

Because platelets are critical for the generation of contractile force, we studied the functionality of platelets in VTE using a flow cytometry-based evaluation of the baseline activity of platelets and their responsiveness to chemical activation with the receptor-activating peptide (TRAP-6). Platelet reactivity was assessed by surface expression of P-selectin and by the ability to bind fibrinogen as a measure of the integrin αIIbβ3 activation. In addition, isolated unstimulated platelets were studied with scanning electron microscopy to assess their baseline functional status reflected by their shape.

**Fig. 5:**
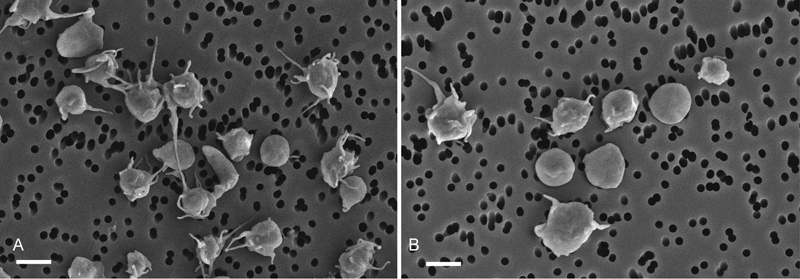
Representative scanning electron micrographs of unstimulated platelets isolated from the blood of a VTE patient ( **A** ) and a healthy donor ( **B** ), showing a higher degree of partial activation of the unstimulated (quiescent) platelets in VTE reflected by the shape change, formation of filopodia, and tendency to aggregate. Magnification bars = 2 µm.

In VTE, platelets were initially partially activated as revealed by the frequently observed shape change and formation of filopodia in platelets from VTE patients (average: 75%) compared with the less common morphologically altered platelets from healthy individuals (average: 21%, *p* < 0.001, *χ* ^2^ test; Table 3 , Fig. 5 ). In contrast to the morphological changes, there was no significant difference in the baseline platelet activation assessed by the levels of P-selectin expression and αIIbβ3 activation in unstimulated platelets ( Table 3 , Supplementary Fig. S1 ). In response to TRAP-6-induced stimulation, platelets from VTE patients had a 1.4-fold lower expression of P-selectin and reduced fibrinogen-binding capacity compared with the TRAP-6-activated normal platelets ( Table 3 , Supplementary Fig. S1 ). Collectively, these results indicate that, in VTE, platelets are continuously activated to a certain extent and have a remarkably reduced responsiveness to a thrombin-like stimulus. It is noteworthy that the ratio of activated versus unstimulated platelets with both fluorescent markers was two- to sixfold higher in healthy donors compared with the VTE patients, suggesting that the VTE platelets are refractory and have a substantially decreased overall activation potential.

**Table 3:** Functional and morphological characterization of platelets isolated from the blood of VTE patients and healthy donors before and after activation with TRAP-6 a

	P-selectin expression b			Fibrinogen-binding capacity b			Shape change c
	Quiescent platelets	TRAP-activated platelets	Activated/quiescent ratio	Quiescent platelets	TRAP-activated platelets	Activated/quiescent ratio	
VTE patients ( *n* = 11)	1.5 ± 0.4	30.0 ± 6.6 *	20	1.3 ± 0.67	41.3 ± 8.4 *	7	75 ± 4% *** (out of 4,211 platelets)
Healthy donors ( *n* = 11)	0.9 ± 0.3	43.0 ± 5.8	44	1.35 ± 0.4	60.2 ± 7.3	42	21 ± 2% (out of 480 platelets)

Abbreviation: VTE, venous thromboembolism.

a TRAP-6 stands for thrombin receptor-activating hexapeptide.

b Numbers (mean ± SEM) represent relative flow cytometry counts (%) for the fractions of platelets bearing antibodies to P-selectin or fluorescently labeled fibrinogen.

c Numbers (mean ± SEM) represent fractions of isolated untreated platelets (%) with shape changes characteristic of platelet activation determined with scanning electron microscopy.

* *p* < 0.05

*** *p* < 0.001.

### Clot Contraction and Clinical Characteristics of VTE Patients

Analysis of clinical data revealed important associations between the changes in contraction of blood clots and the incidence of thromboembolism, the size of the floating part of a thrombus measured with ultrasonography, and duration of VTE symptoms ( Table 4 ). The most significant finding is that the degree and rate of clot contraction were remarkably reduced in patients with PE compared with patients with isolated DVT, while in both populations contraction was still significantly reduced compared with the healthy donors ( Table 4 , Supplementary Figs. S2 S3 S4 ). In DVT patients with a floating head of a thrombus, which is known to be associated with an increased risk of embolization, 24 clot contraction was also significantly suppressed. In patients with acute thrombosis lasting <21 days, shortening of the contraction lag time was observed compared with VTE patients with subacute thrombosis lasting >21 days ( Table 4 ). There were no differences in clot contraction between patients with provoked VTE and those with unprovoked VTE ( Supplementary Table S3 ).

**Table 4:** Blood clot contraction in the clinical subgroups of patients with VTE

Clinical subgroups of VTE patients		Parameters of clot contraction (M ± SEM)			
		Extent of contraction, %	Lag time, s	Average velocity, %/s × 10 ^−3^	AOC, a.u.
Pulmonary embolism	No ( *n* = 31)	35 ± 2	206 ± 16	0.29 ± 0.01	238 ± 13
	Yes ( *n* = 23)	29 ± 2 *	157 ± 21 *	0.23 ± 0.02 *	214 ± 17
Floating part of a thrombus	No ( *n* = 26)	38 ± 2	207 ± 18	0.30 ± 0.01	247 ± 13
	Yes ( *n* = 29)	29 ± 2 **	166 ± 18	0.23 ± 0.02 **	210 ± 14
Duration of symptoms	<21 d ( *n* = 45)	32 ± 2	198 ± 15	0.26 ± 0.01	220 ± 11
	>21 d ( *n* = 10)	36 ± 3	128 ± 19 *	0.29 ± 0.02	264 ± 24 *

Abbreviation: VTE, venous thromboembolism.

* *p* < 0.05

** *p* < 0.01 within clinical subgroups.

## Discussion

### A Summary of the Results and Their Importance

This work is the first systematic and quantitative investigation of clot contraction in VTE patients. It generalizes the potential relevance of clot contraction from arterial thrombosis 18 to venous thrombosis and for the first time points at a possible link between clot contraction and embolization. The main findings of this study are the following. First, venous thrombi undergo intravital contraction or compaction as revealed by the presence of mechanically deformed polyhedral erythrocytes in the ex vivo thrombi. Second, contraction of clots in the blood of VTE patients is significantly reduced compared with healthy donors and the mechanism of this impairment is attributed to platelet exhaustion and refractoriness, as characterized through morphological changes and reduced response to chemical activation. Third, the rate and extent of clot contraction is further reduced in patients with PE compared with isolated DVT, pointing to a potential role of clot contraction in predisposition to embolization. Therefore, these results provide evidence that platelet-driven contraction of venous thrombi occurs in vivo and that variations of the clot size and compactness can potentially modulate the degree of obstruction of a blood vessel as well as the mechanical and fibrinolytic stability of venous thrombi.

### Pathogenic Significance of the Impaired Blood Clot Contraction

There is emerging indirect evidence that the degree and the rate of contraction of clots and thrombi may be an important pathogenic factor that affects the local blood hydrodynamics and the outcome of thrombosis. 4 17 18 25 Although the severity of thrombosis is largely determined by size and location of the occluded vessel, the ability of the clot to contract more or less can largely influence the vessel cross-sectional area and consequently the blood flow through the vessel and the shear rate of the blood. This has implications for the possibility of embolization, where shear rate coupled with the structure of the clot could influence the predisposition to embolize based on either mechanical and/or enzymatic disintegration.

### Platelet Dysfunction as the Main Mechanism of Impaired Blood Clot Contraction in VTE

The reduced clot contraction in VTE is mainly due to platelet dysfunction caused by partial activation of platelets revealed by their morphology ( Fig. 5 , Table 3 ). Based on the surface expression of P-selectin and fibrinogen-binding capacity, we found that platelets in the blood of VTE patients are partially refractory to a thrombin-like activating stimulus and their overall activation potential is significantly smaller than in normal platelets ( Table 3 ). This background platelet activation and exhaustion are likely due to thrombinemia associated with thrombosis, leading to depletion of platelets' energy potential making platelets partially nonfunctional. 26 Contractility is also impaired in platelets with low energy potential because platelets contain ATP-dependent actomyosin machinery that generates contractile forces, 27 28 29 which are propagated through the clot via platelet–fibrin interactions. 30 31 Therefore, disruption in platelet function results in partial contractile inefficiency in the blood of VTE patients. The chronic platelet activation followed by exhaustion seems to be a universal mechanism underlying impaired clot contraction in thrombotic states. 18

**Fig. 6:**
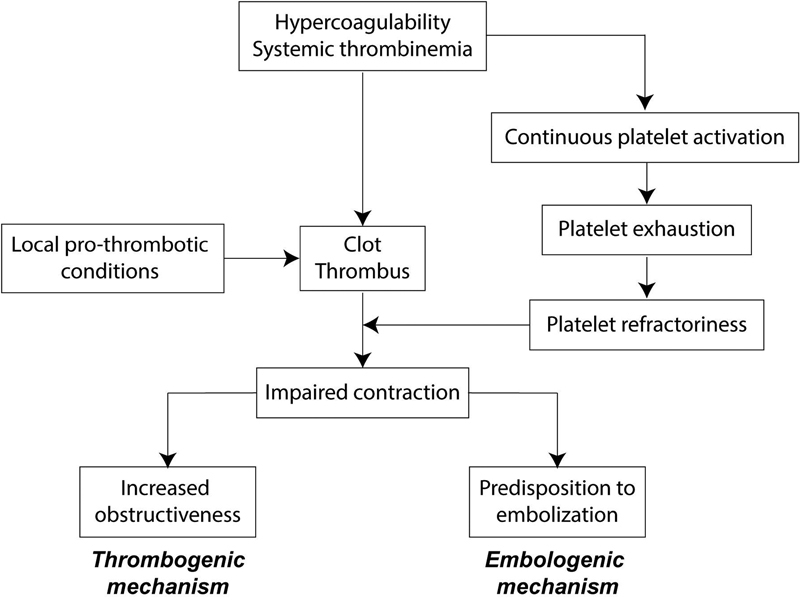
Proposed pathogenic mechanisms related to the impaired clot contraction in venous thromboembolism.

Our concept of the proposed pathogenic mechanisms related to the impaired clot contraction in thrombosis is presented schematically in Fig. 6 .

### Potential Clinical Significance of Blood Clot Contraction in VTE

The most profoundly reduced contraction of blood clots observed in patients with PE ( Table 4 , Supplementary Fig. S2 ) suggests the potential practical importance of the clot contraction assay. If less compacted clots are prone to embolization and the impaired contraction of thrombi may increase the likelihood of thromboembolism, then investigating the dynamics of clot contraction in vitro may complement the existing algorithm for diagnosis of PE. In other words, the laboratory parameters reflecting a remarkable reduction of blood clot contraction in patients with DVT may be considered as an additional sign of a greater risk of embolism.

A larger floating head in venous thrombi is associated with accelerated initiation of clot contraction, indicating faster platelet activation, perhaps due to enhanced local thrombin generation ( Table 4 , Supplementary Fig. S3 ). Therefore, growth of the floating thrombus combined with reduced contractility may be an additional prognostic factor that increases the likelihood of the rupture of the floating part, resulting in thromboembolism.

The clot contraction parameters were influenced by the duration of symptoms where they were more impaired in patients with a shorter duration of symptoms (<21 days) compared with those with symptoms for >21 days ( Table 4 , Supplementary Fig. S4 ). It is likely due to replacement of the older dysfunctional platelets with new functional platelets that partly restore the ability of clots and thrombi to contract. A decrease in fibrinogen level with reduction of inflammation may be another underlying mechanism for normalization of clot contractility. In addition, the synthesis of new prothrombin over time may reduce the extent of thrombinemia and has the potential to normalize platelet activity and partially restore the contractile potential. The increased contraction over time during the course of VTE may comprise a compensatory mechanism for recanalization of the obstructed vessels and may signify a favorable course of the disease.

## Conclusion

Thrombi from VTE patients contained compressed polyhedral erythrocytes, a marker for clot contraction in vivo. The extent and rate of contraction were reduced in clots from the blood of VTE patients compared with healthy controls. The contraction of clots from the blood of patients with PE was significantly impaired compared with that of those with isolated venous thrombosis, suggesting that less compacted thrombi are prone to embolization. The reduced ability of clots to contract correlated with platelet dysfunction. Platelets from the blood of VTE patients were continuously activated as revealed by their shape changes and were refractory to an activating stimulus compared with normal cells. The reduced clot contraction was not apparent in patients 21 days after the acute thrombotic event. The results obtained suggest that contraction of thrombi may be an underappreciated pathogenic mechanism that may affect the course and outcome of VTE. Furthermore, an assay for clot contraction may have hypothetical diagnostic and prognostic value for thromboembolism.

## References

[1] Roger V L Go A S Lloyd-Jones D M Heart disease and stroke statistics - 2012 update: a report from the American Heart Association Circulation 2012 125 01 e2 e220 22179539 10.1161/CIR.0b013e31823ac046 PMC4440543

[2] Bing R Chow V Lau J K Thomas L Kritharides L Ng A CC Prevalence of echocardiography use in patients hospitalized with confirmed acute pulmonary embolism: a real-world observational multicenter study PLoS One 2016 11 12 e0168554 27977781 10.1371/journal.pone.0168554 PMC5158194

[3] Lee J S Moon T Kim T H Deep vein thrombosis in patients with pulmonary embolism: prevalence, clinical significance and outcome Vasc Spec Int 2016 32 04 166 174 10.5758/vsi.2016.32.4.166 PMC5198763 28042556

[4] Wolberg A S Primed to understand fibrinogen in cardiovascular disease Arterioscler Thromb Vasc Biol 2016 36 01 4 6 26700134 10.1161/ATVBAHA.115.306754 PMC4692188

[5] Geddings J E Mackman N Recently identified factors that regulate hemostasis and thrombosis Thromb Haemost 2014 111 04 570 574 24573314 10.1160/TH13-10-0812 PMC4080798

[6] Byrnes J R Wolberg A S New findings on venous thrombogenesis Hamostaseologie 2017 37 01 25 35 27878206 10.5482/HAMO-16-09-0034 PMC5680039

[7] Wolberg A S Aleman M M Leiderman K Machlus K R Procoagulant activity in hemostasis and thrombosis: Virchow's triad revisited Anesth Analg 2012 114 02 275 285 22104070 10.1213/ANE.0b013e31823a088c PMC3264782

[8] von Brühl M L Stark K Steinhart A Monocytes, neutrophils, and platelets cooperate to initiate and propagate venous thrombosis in mice in vivo J Exp Med 2012 209 04 819 835 22451716 10.1084/jem.20112322 PMC3328366

[9] Heestermans M Salloum-Asfar S Salvatori D Role of platelets, neutrophils, and factor XII in spontaneous venous thrombosis in mice Blood 2016 127 21 2630 2637 26932804 10.1182/blood-2015-10-672766 PMC4882807

[10] Walton B L Byrnes J R Wolberg A S Fibrinogen, red blood cells, and factor XIII in venous thrombosis J Thromb Haemost 2015 13 01 S208 S215 26149026 10.1111/jth.12918 PMC5975093

[11] Wakefield T W Strieter R M Wilke C A Venous thrombosis-associated inflammation and attenuation with neutralizing antibodies to cytokines and adhesion molecules Arterioscler Thromb Vasc Biol 1995 15 02 258 268 7749835 10.1161/01.atv.15.2.258

[12] Cines D B Lebedeva T Nagaswami C Clot contraction: compression of erythrocytes into tightly packed polyhedra and redistribution of platelets and fibrin Blood 2014 123 10 1596 1603 24335500 10.1182/blood-2013-08-523860 PMC3945867

[13] Tutwiler V Litvinov R I Lozhkin A P Kinetics and mechanics of clot contraction are governed by the molecular and cellular composition of the blood Blood 2016 127 01 149 159 26603837 10.1182/blood-2015-05-647560 PMC4705605

[14] Carr M E Jr Development of platelet contractile force as a research and clinical measure of platelet function Cell Biochem Biophys 2003 38 01 55 78 12663942 10.1385/CBB:38:1:55

[15] Litvinov R I Weisel J W What is the biological and clinical relevance of fibrin? Semin Thromb Hemost 2016 42 04 333 343 27056152 10.1055/s-0036-1571342 PMC5536100

[16] Kim O V Litvinov R I Alber M S Weisel J W Quantitative structural mechanobiology of platelet-driven blood clot contraction Nat Commun 2017 8 01 1274 29097692 10.1038/s41467-017-00885-x PMC5668372

[17] Muthard R W Diamond S L Blood clots are rapidly assembled hemodynamic sensors: flow arrest triggers intraluminal thrombus contraction Arterioscler Thromb Vasc Biol 2012 32 12 2938 2945 23087356 10.1161/ATVBAHA.112.300312 PMC3586804

[18] Tutwiler V Peshkova A D Andrianova I A Khasanova D R Weisel J W Litvinov R I Contraction of blood clots is impaired in ischemic stroke Arterioscler Thromb Vasc Biol 2017 37 02 271 279 27908894 10.1161/ATVBAHA.116.308622 PMC5269459

[19] Dashkevich N M Vuimo T A Ovsepyan R A Effect of pre-analytical conditions on the thrombodynamics assay Thromb Res 2014 133 03 472 476 24369827 10.1016/j.thromres.2013.12.014

[20] Savelyev V S Chazov E I Gusev E I Russian clinical recommendations for the diagnostics, treatment, and prophylaxis of venous thromboembolism Phlebologie 2010 4(1–2):2 37

[21] Ząbczyk M Sadowski M Zalewski J Undas A Polyhedrocytes in intracoronary thrombi from patients with ST-elevation myocardial infarction Int J Cardiol 2015 179 186 187 25464440 10.1016/j.ijcard.2014.10.004

[22] Leong L Chernysh I N Xu Y Clot stability as a determinant of effective factor VIII replacement in hemophilia A Res Pract Thromb Haemost 2017 1 02 231 241 29713693 10.1002/rth2.12034 PMC5920517

[23] Litvinov R I Khismatullin R R Shakirova A Z Morphological signs of intravital contraction (retraction) of pulmonary thrombotic emboli BioNanoScience 2017 10.1007/s12668-017-0476-1

[24] Blumenberg R M Barton E Gelfand M L Skudder P Brennan J Occult deep venous thrombosis complicating superficial thrombophlebitis J Vasc Surg 1998 27 02 338 343 9510288 10.1016/s0741-5214(98)70364-7

[25] Peshkova A D Le Minh G Tutwiler V Andrianova I A Weisel J W Litvinov R I Activated monocytes enhance platelet-driven contraction of blood clots via tissue factor expression Sci Rep 2017 7 01 5149 28698680 10.1038/s41598-017-05601-9 PMC5506001

[26] Jurk K Jahn U R Van Aken H Platelets in patients with acute ischemic stroke are exhausted and refractory to thrombin, due to cleavage of the seven-transmembrane thrombin receptor (PAR-1) Thromb Haemost 2004 91 02 334 344 14961162 10.1160/TH03-01-0044

[27] Greilich P E Brouse C F Beckham J Jessen M E Martin E J Carr M E Reductions in platelet contractile force correlate with duration of cardiopulmonary bypass and blood loss in patients undergoing cardiac surgery Thromb Res 2002 105 06 523 529 12091054 10.1016/s0049-3848(02)00061-0

[28] Lam W A Chaudhuri O Crow A Mechanics and contraction dynamics of single platelets and implications for clot stiffening Nat Mater 2011 10 01 61 66 21131961 10.1038/nmat2903 PMC3236662

[29] Niederman R Pollard T D Human platelet myosin. II. In vitro assembly and structure of myosin filaments J Cell Biol 1975 67 01 72 92 240861 10.1083/jcb.67.1.72 PMC2109578

[30] Ehrlicher A Hartwig J H Cell mechanics: contracting to stiffness Nat Mater 2011 10 01 12 13 21157494 10.1038/nmat2928

[31] Wufsus A R Rana K Brown A Dorgan J R Liberatore M W Neeves K B Elastic behavior and platelet retraction in low- and high-density fibrin gels Biophys J 2015 108 01 173 183 25564864 10.1016/j.bpj.2014.11.007 PMC4286595

