# Supplementary material for: Reduced Contraction of Blood Clots in Venous Thromboembolism Is a Potential Thrombogenic and Embologenic Mechanism
Source: TH Open. 2018 Mar 28;2(1):e104–15. doi: 10.1055/s-0038-1635572 (PMC6524864; doi:10.1055/s-0038-1635572)
Supplement: Supplementary file 1 — Supplementary Material [file 10-1055-s-0038-1635572-s170022.pdf]

Supplementary Material

Supplementary Materials and Methods

Patients

The total cohort of venous thromboembolism (VTE) patients admitted to the Department of Vascular Surgery of the Interregional Clinical Diagnostic Center (Kazan, Russian Federation) during 2014–2016 comprised 554 individuals of which only 55 (10%) were enrolled in the study based on the following inclusion criteria (see the flowchart below):

- 18-year-old or older with a written informed consent.
- DVT was verified by duplex ultrasonography of the vessels.
- Location of thrombosis: a popliteal vein and a more proximal vein.

The exclusion criteria were as follows:

- Pregnant women and nursing mothers.
- Active cancer.
- Active liver disease (transaminase levels two times or higher than normal).
- Bacterial endocarditis.
- Uncontrolled hypertension (BP > 180/100 mm Hg).
- Platelet count <100,000/µL.
- Hemoglobin <9 g/L.
- Creatinine clearance <25 mL/min.
- Active bleeding or high risk of bleeding.
- *Patients were also excluded if they received heparin (unfractionated or low-molecular-weight) or a vitamin K antagonist, antiplatelet drugs (including NSAIDs), inhibitors of cytochrome P-450, and pro- or antifibrinolytics for at least 1 month prior to the examination.*

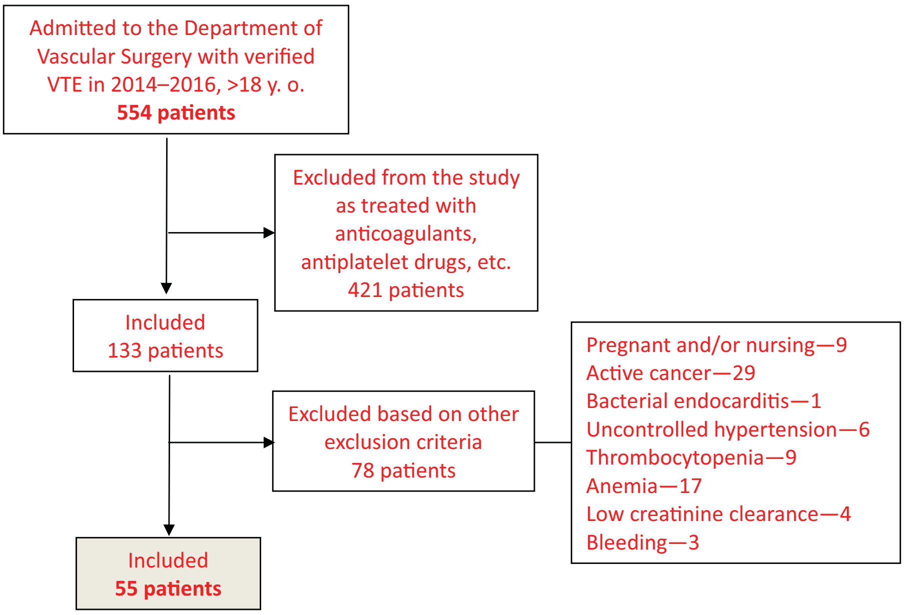

Flowchart of the study.

Table S1 Basic demographic and clinical characteristics of all VTE patients admitted to the ER during 2014–2016 compared with those included in the study based on the inclusion and exclusion criteria

Characteristics	Complete cohort ($n = 554$)	Included in the study ($n = 55$)	Statistical significance[a]
Gender (males, %)	62% (343)	67% (37)	$p = 0.47$
Age (range, y)	18–81	23–78	–
Incidence of PE (%)	31% (172)	42% (23)	$p = 0.16$
Thrombectomy (%)	10% (55)	16% (9)	$p = 0.13$
Patients with floating part of thrombus (%)	42% (227)	53% (29)	$p = 0.092$
Provoked VTE (%)	21% (116)	15% (8)	$p = 0.26$

[a]Chi-square test.

Table S2 Clot contraction parameters in VTE patients and healthy individuals

	VTE patients ($n = 55$)	Healthy subjects ($n = 60$)
Extent of contraction, %	$33 \pm 1^{***}$	48 ± 1
Lag time, s	$185 \pm 13^{***}$	108 ± 9
Average velocity, %/s $\times 10^{-3}$	$0.26 \pm 0.01^{***}$	0.4 ± 0.01
Area over the curve, a.u.	$228 \pm 10^{***}$	371 ± 10

$^{***}p < 0.0001$.

Table S3 Clot contraction parameters in patients with unprovoked and provoked VTE

	Unprovoked VTE ($n = 47$)	Provoked VTE ($n = 8$)
Extent of contraction, %	33 ± 2	32 ± 3
Lag time, s	182 ± 14	198 ± 32
Average velocity, %/s $\times 10^{-3}$	0.26 ± 0.01	0.25 ± 0.02
AOC, a.u.	230 ± 11	217 ± 23

Note: $p > 0.05$ for all compared means.

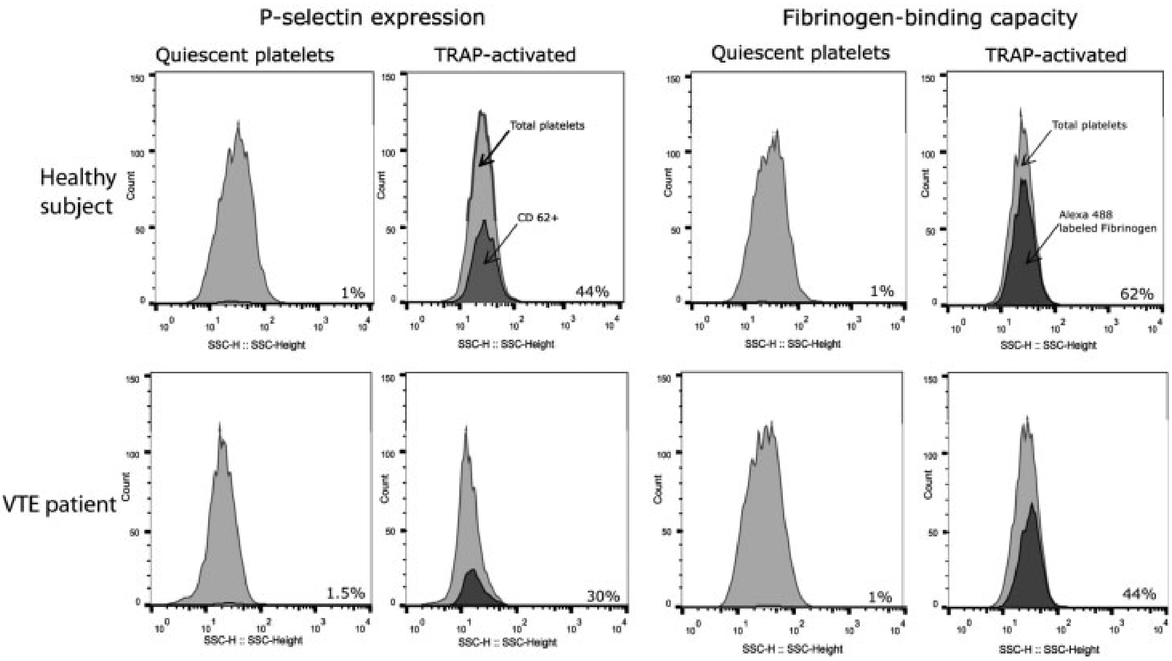

Fig. S1 Representative raw data from flow cytometry of platelets isolated from the blood of a healthy donor and a VTE patient under various experimental conditions. The platelets were incubated with either antihuman CD62 phycoerythrin-labeled antibodies or Alexa fluor 488-labeled human fibrinogen before and after activation with thrombin receptor-activating peptide (TRAP-6). Each plot represents the peak of counts for a fraction of fluorescing platelets superimposed on the peak of total platelet counts (around 5,000 total counts each). Numbers (%) represent a portion of the fluorescing platelets.

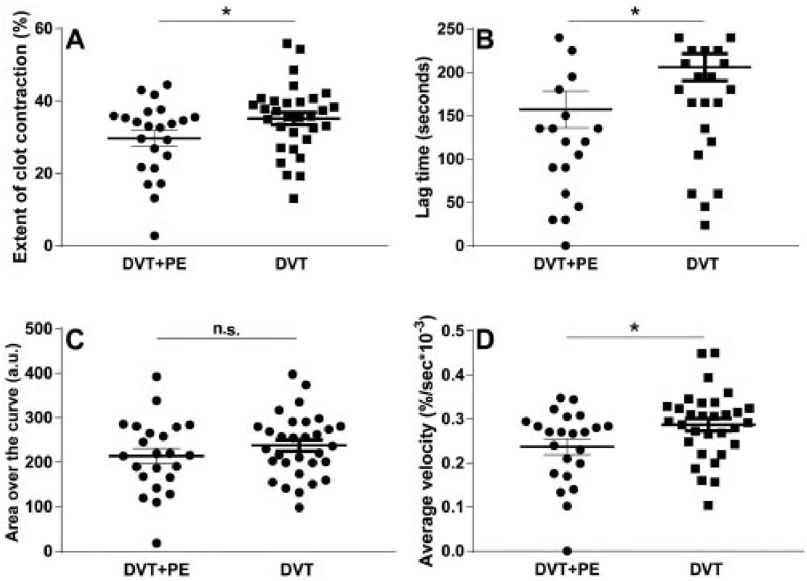

Fig. S2 Clot contraction parameters of clots made from the blood of patients with DVT and PE ($n = 31$) versus DVT alone ($n = 23$). (**A**) extent of clot contraction, (**B**) lag time, (**C**) area over the curve, (**D**) average velocity.

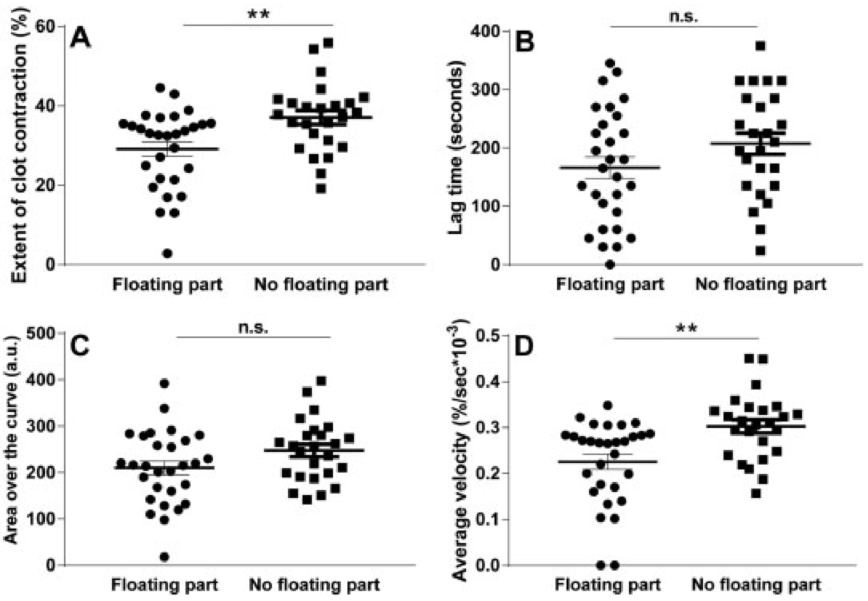

Fig. S3 Clot contraction parameters of clots made from the blood of DVT patients with ($n = 29$) and without ($n = 26$) a floating part of a thrombus. (**A**) Extent of clot contraction, (**B**) lag time, (**C**) area over the curve, (**D**) average velocity.

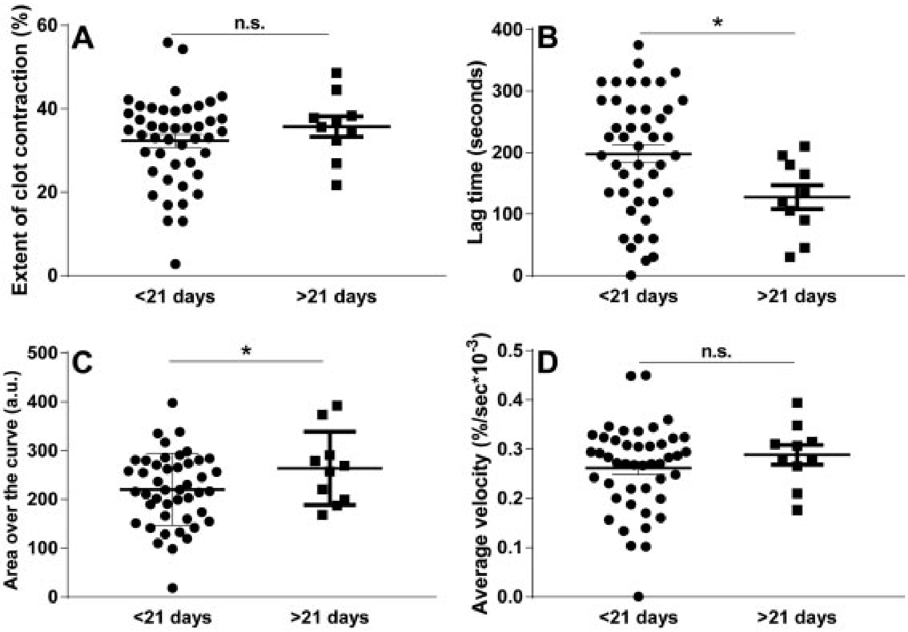

Fig. S4 Clot contraction parameters of clots made from the blood of DVT patients with duration of symptoms <21 days ($n = 45$) and >21 days ($n = 10$). (**A**) Extent of clot contraction, (**B**) lag time, (**C**) area over the curve, (**D**) average velocity.
